# Nocturnal behavioral patterns of African ungulates in zoos

**DOI:** 10.1002/ece3.10777

**Published:** 2023-12-03

**Authors:** Jennifer Gübert, Gaby Schneider, Max Hahn‐Klimroth, Paul W. Dierkes

**Affiliations:** ^1^ Faculty of Biological Sciences Goethe University Frankfurt Frankfurt Germany; ^2^ Institute of Mathematics Goethe University Frankfurt Frankfurt Germany; ^3^ Faculty of Computer Sciences TU Dortmund University Dortmund Germany

**Keywords:** African ungulates, behavioral rhythms, ecology of savannah animals, nocturnal behavior, REM sleep position, zoo animals

## Abstract

Currently, most studies on ungulates' behavior are conducted during the daylight hours, but their nocturnal behavior patterns differ from those shown during day. Therefore, it is necessary to observe ungulates' behavior also overnight. Detailed analyses of nocturnal behavior have only been conducted for very prominent ungulates such as Giraffes (*Giraffa camelopardalis*), African Elephants (*Loxodonta africana*), or livestock (e.g., domesticated cattle, sheep, or pigs), and the nocturnal rhythms exhibited by many ungulates remain unknown. In the present study, the nocturnal rhythms of 192 individuals of 18 ungulate species from 20 European zoos are studied with respect to the behavioral positions standing, lying—head up, and lying—head down (the typical REM sleep position). Differences between individuals of different age were found, but no differences with respect to the sex were seen. Most species showed a significant increase in the proportion of lying during the night. In addition, the time between two events of “lying down” was studied in detail. A high degree of rhythmicity with respect to this quantity was found in all species. The proportion of lying in such a period was greater in Artiodactyla than in Perissodactyla, and greater in juveniles than in adults.

## Graphical abstract

In the present study, the nocturnal activity patterns of 192 individuals of 18 ungulate species from 20 European zoos are studied with respect to the behavioral positions standing, lying—head up, and lying—head down (the typical REM sleep position). Most species showed a significant increase in the proportion of lying during the night. In addition, a high degree of rhythmicity with respect to the time between two events of “lying down” was found in all species. Significant differences were found between individuals of the orders Artiodactyla and Perissodactyla, as well as individuals of different age groups.

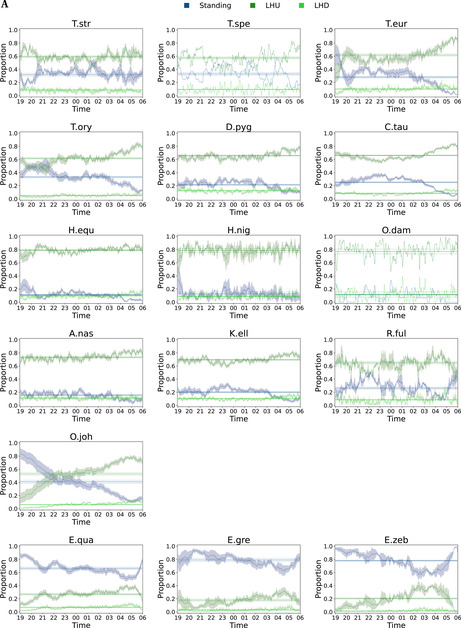

## 1 INTRODUCTION

The description and analysis of animal behavior is a central element of behavioral ecology. There are over 250 Artiodactyla and Perissodactyla (IUCN, 2022), and these ungulates are clearly among the characteristic animals of the African landscape. It is, therefore, perhaps not surprising that some of the most widely kept zoo animals belong to these orders (Rose & Robert, 2013). While it is not only a fundamental element of behavioral biology to gain knowledge about the behavior of such a prominent group of animals, insights into the behavior of a species can also help in animal management and husbandry (Rose & Riley, 2021). In fact, knowledge of behavioral patterns can improve husbandry conditions, which in turn can improve animal welfare (Berger, 2010; Brando & Buchanan‐Smith, 2018; Rose & Riley, 2021; Walsh et al., 2019).

There is extensive knowledge about the behavior and especially the rhythms displayed during the daylight hours of various ungulates like, for example, European Bisons (*Bos bonasus*) (Caboń‐Raczyńska et al., 1983), Gaurs (*Bos gaurus*) (Manjrekar et al., 2017), Hippotraginae (Packard et al., 2014), Plains Zebras (*Equus quagga*) (Reta & Solomon, 2014), Water Deer (*Hydropotes inermis*) (Zhang, 2000), and Gerenuks (*Litocranius walleri*) (Leuthold & Leuthold, 1978). The categories of “activity” and “rest” are the most prominent behavioral states measured to study rhythms (Merrow et al., 2005). There are four main temporal partitioning strategies that differentiate activity patterns: nocturnal, diurnal, cathemeral, and crepuscular (Bennie et al., 2014). Most ungulates of the orders Perissodactyla and Artiodactyla are diurnal or crepuscular (Bennie et al., 2014), that is, behavior patterns during daylight and during night differ (Davimes et al., 2018; Gravett et al., 2017; Wu et al., 2018). In particular, sleeping patterns are shifted into the night or dusk in many ungulates (Bennie et al., 2014; Gravett et al., 2017; Wu et al., 2018), and, for instance, Arabian Oryx (*Oryx leucoryx*) shift their sleeping patterns even further into the night during the colder months (Davimes et al., 2018). Therefore, to fully understand the behavior of a species, it is necessary to also analyze its nocturnal behavior. Of course, nocturnal behavior is well studied for some very prominent species, such as Giraffes (*Giraffa camelopardalis*) (Burger et al., 2020; Sicks, 2016; Tobler & Schwierin, 1996) and African Elephants (*Loxodonta africana*) (Gravett et al., 2017), or for various farm animals (Greening & McBride, 2022; Ruckebusch, 1972; Ternman et al., 2014). However, to the best of our knowledge, there is much less literature on the nocturnal behavior of many other ungulates.

Many challenges arise in the analysis of nocturnal behavior when animals are observed in their natural habitat. It is much more accessible to observe the nocturnal behavior of zoo animals (Ryder & Feistner, 1995). Observations in zoos provide an excellent opportunity to generate vast knowledge about animal behavior (Hollén & Manser, 2007; Melfi & Feistner, 2002; Rees, 2023), as zoos provide consistent and better access to animals and easier conditions for data collection (Ryder & Feistner, 1995). The latter is a requirement for understanding animal behavior on much more data than could be recorded in the wild. However, the ecology of zoo animals can differ from the ecology of the wild living conspecifics. Thus, certain aspects of the activity budgets vary. Nevertheless, it is well known that zoo animals and their wild conspecifics are comparable with respect to a variety of characteristics (Burger et al., 2020). Therefore, studies conducted with zoo animals help us to learn about the species' behavior in the wild (Rees, 2023). Especially in zoos, video recordings are a good tool to study nocturnal behavior because the observation method is noninvasive and does not cause behavioral changes by disturbing the observed animals.

### 1.1 Aims and scope

The current study is based on the results of a recent study investigating the basic characteristics of nocturnal behavior in ungulates (Gubert et al., 2023). The previous contribution examined the factors that influence the behavioral poses standing, lying—head up (LHU), and lying—head down (LHD). LHD is the typical REM (rapid eye movement) sleep posture which can be used to estimate REM sleep noninvasively (El Allali et al., 2022; Greening & McBride, 2022; Lyamin et al., 2021; Ternman et al., 2014). More specifically, the previous study identified the main factors influencing the activity budget and the number of phases per night of these behaviors. Age, body size, and the digestion type were found to have a strong influence (Gubert et al., 2023). However, the activity budget and the number of phases of a behavior during night give a fundamental, though very basic, description of behavior.

The current study takes a much finer look at behavior patterns, also distinguishing the behavioral poses standing, LHU, and LHD. We consider this contribution important to understand ungulates' nocturnal behavior in more detail. Its results may represent an important baseline for the assessment of animal welfare, because deviations from typical behavioral patterns can be an indication of stress (Sicks, 2016). Thus, the aim of the current study is to describe the following aspects of nocturnal behavior for a wide range of species. First, the development of the three behavioral states occurring during the night is presented on a species level, and different clusters of species regarding their behavioral patterns are found. Second, we investigate properties of the lying‐standing cycle of every individual. More precisely, we study the mean and the distribution of the time between two consecutive events of lying down, which will be called a *lying cycle* (LC) in analogy to the classical sleep cycle investigated in humans (Dement & Kleitman, 1957). Third, within each LC, the fraction of lying and standing behavior is investigated, particularly with respect to age differences. Finally, the time spent in the REM sleep position (LHD) is studied in more detail. For each individual, we study the number of LHD phases as well as the proportion of LHD in each lying phase.

## 2 MATERIALS AND METHODS

### 2.1 Study population

Overall, the nocturnal behavior of 192 individuals from 18 species was investigated based on 9156 single night recordings. The following species were included: Greater Kudu (*Tragelaphus strepsiceros*), Sitatunga (*T. spekii*), Bongo (*T. eurycerus*), Common Eland (*T. oryx*), African Buffalo (*Syncerus caffer*), distinguished into the subspecies African Forest Buffalo (*S. caffer nanus*) and African Savannah Buffalo (*S. caffer caffer*), Blesbok (*Damaliscus pygargus*), Common Wildebeest (*Connochaetes taurinus*), Roan Antelope (*Hippotragus equinus*), Sable Antelope (*H. niger*), Scimitar‐horned Oryx (*Oryx dammah*), Addax (*Addax nasomaculatus*), Waterbuck (*Kobus ellipsiprymnus*), Mountain Reedbuck (*Redunca fulvorufula*), Okapi (*Okapia johnstoni*), Plains Zebra (*Equus quagga*), Grevy's Zebra (*E. grevyi*) and Mountain Zebra (*E. zebra*). Data were collected from the end of September until the beginning of May (during the colder season) in the years 2017 to 2021 in 20 EAZA zoos. The number of recorded nights per individual ranged from 9 to 185, with a median of 47. The number of recorded individuals per species ranged from 1 Sitatunga to 32 Plains Zebras. We distinguished between male and female individuals as well as between young, subadult, and adult individuals. The age categories are defined via the time of weaning (from young to subadult) and the sexual maturity (from subadult to adult) of the species. The proportion of individuals in the different age categories and different sex are given in the supplemental material.

### 2.2 Ethogram

The ethogram is defined as in the study by Gubert et al. (2023) and can be found in Table 1. The two main behaviors are standing and lying. Lying is further divided into lying—head up (LHU) and lying—head down (LHD). LHD describes the typical REM (rapid eye movement) sleep posture of the studied species and can be used to estimate REM sleep. Although measuring LHD yields only an approximation to REM sleep, previous studies prove that LHD is a reliable indicator. Its validity increases, in particular, if the REM sleep phases become longer (El Allali et al., 2022; Greening & McBride, 2022; Seeber et al., 2012; Ternman et al., 2014; Zizkova et al., 2013). If no animal is present on the recording, the category out of view (Out) is assigned.

**TABLE 1:** Ethogram used in the study as defined by Gubert et al. (2023).

	Behavior	Description
Standing	Standing	The animal stands in an upright position. Other behaviors like feeding, resting, walking, or ruminating can occur simultaneously
Lying	Lying—head up (LHU)	The animal is in a sternal recumbency with the trunk touching the ground. Its head is lifted
	Lying—head down (LHD)	*Artiodactyla*: The animal is in a sternal recumbency with the trunk touching the ground (like in Lying—head up) but its head is resting on the ground. *Perissodactyla*: The animal is lying in a lateral recumbency

### 2.3 Lying cycles and lying fraction

**FIGURE 1:**
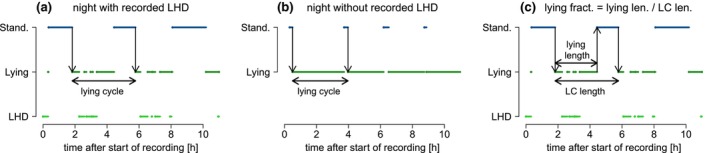
The definition of lying cycles (LC) in nights with recorded lying—head down (LHD) (a) and without recorded LHD (b). (c) visualizes the definition of the lying fraction (LF).

To study rhythms in the described behavioral poses, lying cycles (LC) and the lying fraction were used. LCs are defined as the periods starting at the first lying phase observed after a standing phase to the next lying phase observed after the following standing phase (Figure 1a,b), where only LCs without Out in this period were used in the analysis. For every LC, the fraction of lying (LF) in this cycle was calculated and denoted by LF (Figure 1c). For example, if the duration of lying was 30 min in a lying cycle that had a total length of 2 h, the LF was 0.25.

### 2.4 Data recording and data processing

Data were collected by video recordings with night vision cameras with built‐in infrared emitters (Lupus LE139HD or Lupus LE338HD with the recording device LUPUSTEC LE800HD or TECHNAXX PRO HD 720P). The frame rate is 1 fps and the resolution ranges from 704 × 576 px to 1920 × 1080 px. The cameras were installed in the stables of animals in 20 EAZA zoos in Germany (Zoologische Gärten Berlin (Tierpark and Zoo), Zoo Vivarium Darmstadt, Zoo Dortmund, Zoo Duisburg, Zoo Frankfurt, Zoom Erlebniswelt Gelsenkirchen, Erlebnis‐Zoo Hannover, Zoo Heidelberg, Kölner Zoo, Zoo Krefeld, Opel‐Zoo Kronberg, Zoo Landau in der Pfalz, Zoo Leipzig, Allwetterzoo Münster, Zoo Neuwied, Zoo Osnabrück, Zoologischer Garten Schwerin, Der Grüne Zoo Wuppertal) and the Netherlands (Königlicher Burgers Zoo Arnheim). Data collection took place from the end of September until the beginning of May in the years 2017 to 2021 during night, with a “night” being defined as the time from 7 p.m. to 6 a.m.

To evaluate the nights, the deep learning‐based software package BOVIDS (Behavioral Observations by Videos and Images using Deep Learning Software) was used (Gubert et al., 2022). As usual in machine learning tasks, the underlying neural networks need to be trained by manually annotated data (the *training set*) and the accuracy of the system needs to be tested by comparing the predictions against manually annotated data (the *testing data*). To this end, 517 of the 9156 recorded nights were evaluated manually with the open source software BORIS (Behavioral Observation Research Interactive Software), version 7.7.3 (Friard & Gamba, 2016). Here, a continuous sampling was used such that the behavior of each animal is known at every second (Martin & Bateson, 2015). All other nights were annotated using BOVIDS. BOVIDS achieved average f‐scores of 0.992 ± 0.003 (lying), and 0.956 ± 0.006 (LHD) on the unseen testing data. Detailed information on the performance per individual on the testing set is given in the Appendix S1. The software package BOVIDS applies a set of postprocessing rules to achieve high classification accuracy (Gubert et al., 2022; Hahn‐Klimroth et al., 2021). Most importantly, standing and lying sequences shorter than 5 min and LHD sequences shorter than 35 s are discarded. In addition, on the analyzed nights, recordings where an animal is not present for at least 20% of the time and recordings with at least three occurrences of Out are discarded. A total of 9156 nights with 100,716 h were evaluated for standing and lying discrimination, with a median number of recorded nights of 47 per individual. The quartile deviation amounts to 11.5. On a subset of the data, lying was further distinguished into LHU and LHD. This reduced dataset consisted of 6226 nights from 129 individuals. Detailed information about the sample sizes are provided in the Appendix S1.

After discarding nights as described above, 733 nights out of 9156 nights contained sequences of Out. To study lying cycles, that is, periods between two consecutive events of lying after standing, and the lying fraction (see Section 2.2), the following preprocessing was performed. This preprocessing was not performed for the description of standing and lying during night in Section 3.1. First, Out periods occurring at the beginning (or the end) of a night were simply discarded by starting (ending) the night at its first (or last) classifiable event. Second, a sequence of “standing—Out—standing” in which the out period was shorter than 30 min was merged into a standing period. Third, in a sequence of “lying—Out—standing” or “standing—Out—lying” in which the Out period was shorter than 30 min, the out period was considered as standing and thus merged with the standing period. This preprocessing reduced the number of nights with Out periods to only 297 out of a total of 9156 nights. For the analysis of lying cycles we then included only cycles without remaining Out periods.

### 2.5 Statistical methods

We used R to conduct standard nonparametric procedures as the Wilcoxon‐, and Kruskal‐Wallis tests for statistical comparisons (R Core Team, 2022). The three levels of significance 5%, 1% and 0.1% are indicated by *, **, and ***, respectively. For data preparation the Python programming language and the pandas library (Pandas Team, 2022) were used. Visualizations were done in R and matplotlib (Hunter, 2007).

To classify the increase and decrease of the lying proportion during the night for each species (Section 3.1), we considered all adult individuals of each species separately. For each individual, we then estimated the slopes of the lying proportion in the first (from 19:00 to 00:30) and in the second half (from 00:30 to 06:00) of the nights using standard linear regression. As a simple classification heuristic, we tested the slopes of all individuals per species for systematic deviations from zero in each part of the night, as well as for differences between the first and second part of the night using *t*‐tests for all species with at least three adult individuals. Species with, for example, nonsignificant (*p* > .05) results for the first part of the night, and positive slopes with *p* < .05 in the second part of the night also showed significant differences in slopes between first and second part of the night and were classified in the same class. For the other classes, we proceeded analogously.

In order to describe the distribution of lying cycles (LCs) (Section 3.2), we considered the distribution of LCs for every individual separately and derived its mean and standard deviation. We also derived the 1% quantile, Q01, of the LC distribution for every individual. The median of these Q01 values, MQ01, for the single adult animals of the respective species was then used as a reference for the respective species, that is, we then derived for every individual the percentage of LCs below the respective MQ01 reference value.

Throughout the paper, qualitative results are illustrated for exemplary animals when results across animals are comparable. Differences, that is, between means or other parameters, are then illustrated in overview graphs that show the estimated parameters as a function of species, sex and age group simultaneously.

While the dataset contains many individuals observed over a high number of nights and a large number of different species, one should note that the data are highly unbalanced. Therefore, possibilities of rigorous statistical analysis such as, for example, highly dimensional ANOVA or even phylogenetic comparative methods (O'Meara, 2012), were limited for the present dataset. That is, some species showed only few observations, not every age group and sex was observed for every species, and as common when studying ungulates, the ratio between male and female individuals was small. If one species' sample contained no male animals and another species no female animals, differences could be attributed to either sex or species for example. Therefore, we refrained from a statistical comparison between all species, focusing rather on graphical comparison.

In order to compare different age groups, we used species standardization, that is, we standardized, for example, the LF with the mean and standard deviation of LFs of all individuals of the respective species. That is, for each individual, the species' mean was subtracted from the respective quantity, and the result was then divided by the species' estimated standard deviation. In this way, the measurement of every animal indicates the standardized deviation from the mean in the respective species, such that measurements between different species are comparable, which allows a comparison between age groups or between males and females.

In order to investigate the relation between the total LHD duration within a lying phase and the lying duration, we applied censored linear regression in the classical Tobit model using the R package censReg (Henningsen, 2010, 2017) in order to take into account that the total LHD duration cannot be negative. Analogously, the relation between the lying duration and the number of phases LHD per lying phase was also investigated with censored linear regression. The censored linear regression applied here uses the same assumptions as ordinary linear regression, except for assuming that the total LHD duration is conditioned to be non‐negative, that is, censored at zero. The parameters of the regression line are derived by maximum likelihood estimation, but due to the censoring, maximum likelihood estimates do not have a closed form but need to be obtained via numerical maximization.

## 3 RESULTS

### 3.1 Proportion of the behavioral poses

**FIGURE 2:**
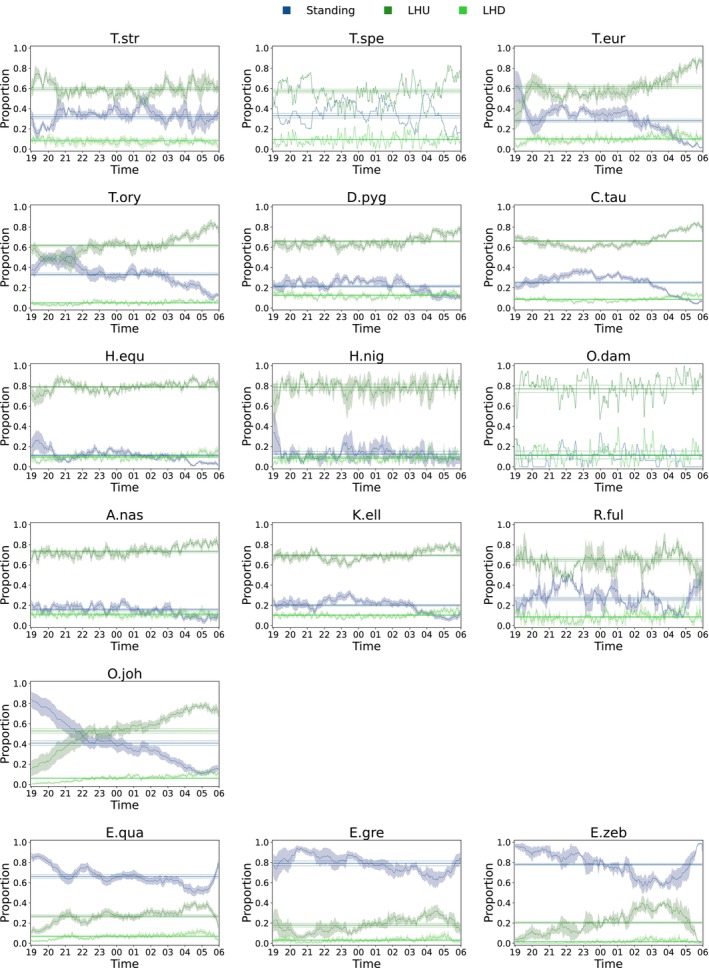
Distribution of the mean nocturnal behavior of the adult individuals per species for those species on whose recordings lying was distinguished into lying—head up (LHU) and lying—head down (LHD). The *y*‐axis reports the proportion of the behavioral pose and the area around the curve visualizes the SEM over all individuals of a species. The horizontal lines mark the mean (solid) and the standard deviation (dotted) of the behavior in a complete night. The exact sample sizes are given in the Appendix S1.

**FIGURE 3:**
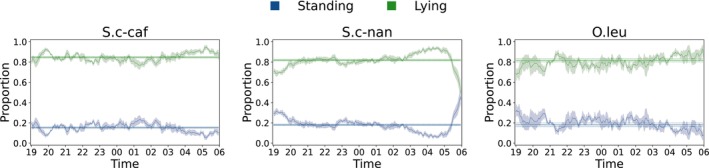
Distribution of the mean nocturnal behavior of the adult individuals per species for those species in which LHD was not evaluated. The *y*‐axis reports the proportion of the behavioral pose and the area around the curve visualizes the SEM over all individuals of a species. The horizontal lines mark the mean (solid) and the standard deviation (dotted) of the behavior in a complete night. The exact sample sizes are given in the Appendix S1.

The first set of results is a description of the behavioral poses' proportions. Figures 2 and 3 reports each species' mean of the proportion per behavioral pose over a night. The figure visualizes the change in the proportion of behavioral poses as the night progresses.

**FIGURE 4:**
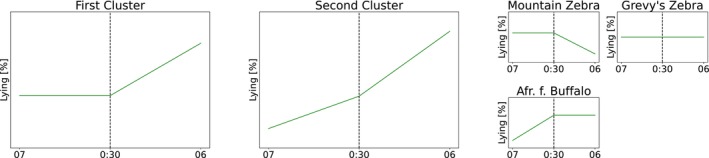
A schematic visualization of two clusters (and three exceptions) of the change in the proportion lying during the night. The first cluster contains of the following 12 species: Addax, African Savannah Buffalo, Arabian Oryx, Blesbok, Bongo, Greater Kudu, Mountain Reedbuck, Roan Antelope, Sable Antelope, Scimitar‐horned Oryx, Sitatunga, and Waterbuck. Those species show an increase in the lying proportion only during the second part of the night. The second cluster comprises four species: Okapi, Plains Zebra, Common Eland and Common Wildebeest. Those species show an increase in the lying proportion in both parts of the night. The formation of clusters is described in Section 2.5 in detail.

The three zebra species have a much higher proportion of standing compared to all Artiodactyla. In the following, the change of the proportion of lying over the night is analyzed in more detail. More precisely, it is possible to cluster the single species into two clusters with respect to the change in the proportion of lying in the first and the second part of the night (see Figure 4).

Details of this clustering are given in Section 2.5. In the first cluster, the lying proportion shows no particular change in the first part of the night, but a significant increase in the second part of the night. This cluster consists of 12 species (Addax, African Savannah Buffalo, Arabian Oryx, Blesbok, Bongo, Greater Kudu, Mountain Reedbuck (†), Roan Antelope, Sable Antelope (†), Scimitar‐horned Oryx, Sitatunga (†), and Waterbuck). The species marked with a † could not be tested for statistical significance due to low sample sizes, but these species show a similar pattern. The species in the second cluster exhibit an increase in the lying proportion in both parts of the night, this cluster consists of four species (Okapi, Plains Zebra, Common Eland and Common Wildebeest). Finally, three species showed a different pattern and did not fit into one of the two clusters. Mountain Zebras showed no particular change in the first part of the night, but the lying fraction decreased in the second part. Grevy's Zebras showed no change in either part, and the African Forest Buffaloes showed an increase in the first part, but no increase in the second part. Note that, also for the three species that did not fit into the clusters, an increase in the second part of the night was visible until the last 1–2 h. This means that up to the last 2 h of the recording, Grevy's Zebras could be sorted into the first cluster and African Forest Buffaloes might be part of the second cluster.

Regarding the more fine‐grained view on LHD it can be observed in Figure 2 that LHD is shown throughout the night in every species. This indicates that LHD is shown regularly in any sufficiently long lying phase. This will be presented in detail in Section 3.3.

**FIGURE 5:**
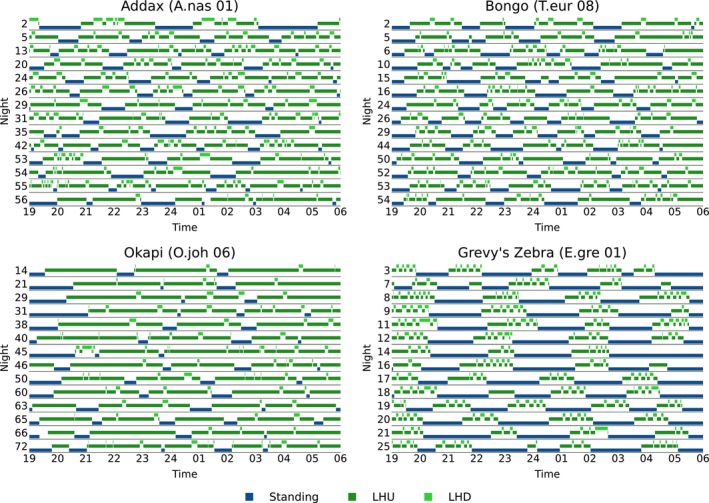
Exemplary representation of the nocturnal behavior of four adult individuals of the species Addax, Bongo, Okapi, and Grevy's Zebra. The behavioral poses standing (blue), lying—head up (dark green), and lying—head down (light green) are shown. For each individual, 14 randomly chosen nights are displayed.

While it is beyond the scope of the current study to present the behavior of every single individual, Figure 5 exemplarily reports the nocturnal behavior of four adult individuals in detail. To get a good impression about fluctuations between nights, but without reporting too much details, 14 nights (or 2 weeks) of recorded data are shown. Those nights were chosen randomly to ensure that not only consecutive nights following a certain (artificial) structure are presented. Moreover, a male Addax and a female Bongo are chosen as representatives of the family Bovidae, a female Okapi as a representative of the family Giraffidae and, finally, a male Grevy's Zebra represents the family Equidae within the order Perissodactyla. Those species represent the three families studied in this contribution. The examples underline that there are no severe changes in the analyzed behavioral poses in different nights of one individual. In particular, the examples show that the number and the length of the standing and lying phases of one individual do not seem to change largely in their pattern, and their typical length does not vary strongly between different nights. Finally, the patterns described above of the proportion of lying are visible in these representatives for the corresponding species.

### 3.2 Lying cycles (LC) and lying fraction (LF)

**FIGURE 6:**
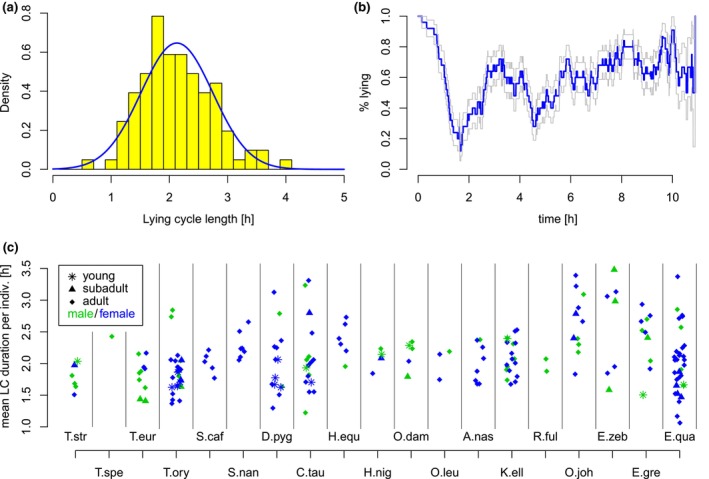
Lying cycles (LC) can occur rhythmically. (a) Distribution of LC lengths are typically symmetric. Here: LC distribution of a female adult Common Eland. (b) Average percentage of lying and standing during all nights of this individual, aligned at the start of the first fully observed lying cycle (blue: proportion, gray: 95%‐confidence band). (c) Mean LC duration for each animal.

In the previous subsection, the basic patterns of the proportion of lying were presented. In the following, a more detailed view on rhythms occurring over the night is given by studying lying cycles (LC). Around 86% of the analyzed individuals showed a regular distribution of LC durations, which could be described approximately by a normal distribution. An example of an adult Common Eland is given in Figure 6a. Accordingly, this regularity was often reflected in a certain rhythmic change of lying and standing periods when aligning the behavior at the start of the first lying cycle (Figure 6b).

The mean LC duration per individual is reported in Figure 6c. The mean LC duration per species ranged between 1.8 h in Greater Kudu and 2.6 h in Okapi, with a mean of 2.14 h and a standard deviation of 0.24 h across all species. This indicates relatively high comparability between species in the mean LC duration. In addition, no considerable difference could be observed as a function of sex or age.

**FIGURE 7:**
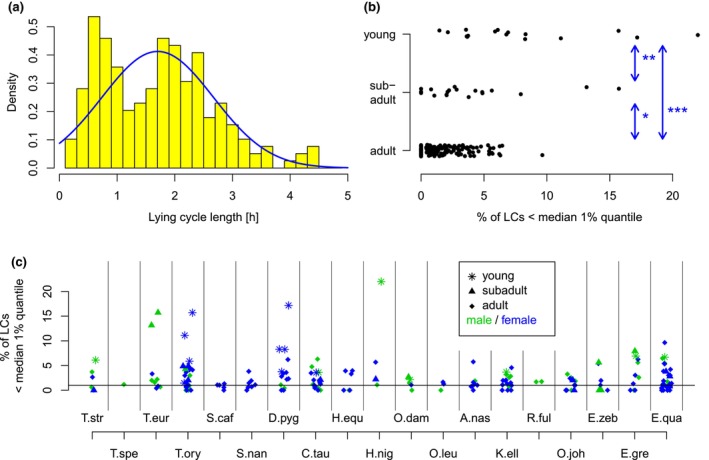
The durations of lying cycles (LC) are less regular in young animals, showing a high degree of short LCs. (a) Distribution of LC lengths in a female young Common Wildebeest. (b, c) Percentage of LCs per individual shorter than median 1% quantile in single adults in the respective species, as a function of age (b) and species and age (c). Stars indicate statistical significance on the 5% (*), 1% (**) and 0.1% (***)‐level.

In around 14% of the analyzed individuals severe deviations of LC lengths from a symmetric distribution could be observed (see Figure 7a; example of a young Common Wildebeest). In such cases, animals showed a high degree of very short LCs as compared to the typical LC lengths in the respective species, and such distributions could typically be observed in younger animals. Indeed, 62.5% of the juvenile individuals show a large proportion of such short LC lengths while this is only the case for 20% of the subadult and 7.7% of the adult individuals. In order to quantify this effect, we calculated the 1% quantile of the LC distribution for all adult individuals being stalled as the only individual in a box, and then used the medians of these 1% quantiles across each species as references. As the normalization is performed for each species, no species specific differences can be observed. This normalization allows us to compare the proportion of short phases of young, subadult and adult individuals for different species. A visualization is given in Figure 7b. Differences between age groups in the percentage of short LCs were statistically significant (*p* < .01 for young vs. subadult, *p* < .03 for subadult vs. adult, *p* < .0001 for young vs. adult, Wilcoxon rank sum tests). Interestingly, these differences were not reflected in systematic age differences in the mean LC duration (see Figure 6c). Figure 7c shows for each individual the percentage of LCs shorter than this reference. The individuals are distinguished by their species, their sex and their age. Adult individuals range around the 1% quantile (horizontal line), while some strong deviations up to more than 20% of short LCs can be observed, particularly in young and subadult individuals which are indicated by stars and triangles, respectively.

**FIGURE 8:**
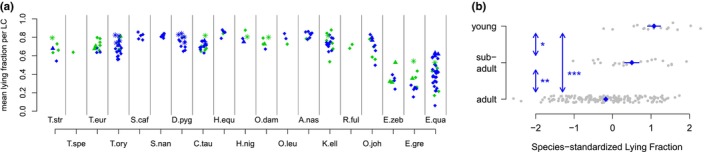
Length of lying relative to the lying cycle (LC) duration tends to be longer in young animals. (a) LF as a function of the age group, standardized with the mean and standard deviation of the LF in each respective species. Blue diamonds and lines indicate mean ± SEM. (b) Mean LFs per animal as a function of the species, age and sex. Point characters and colors as in Figure 6.

The fraction of lying (LF, see Figure 1c for definition) during an LC differed considerably across species, ranging between a mean of about 31% in Grevy's Zebras up to a mean of about 85% in Roan Antelopes (Figure 8a). Visual inspection of the figure leads to two clusters of species. On the one hand, there are the three Perissodactyla (35.8% ± 3.8%) and on the other hand, the studied Artiodactyla (74.6% ± 6.1%). The mean lying fractions of Perissodactyla and Artiodactyla were highly separated: While only 6.2% of all Perissodactyla showed a mean lying fraction above 60%, <7% of all Artiodactyla showed a mean lying fraction below 60% (Figure 8a). Furthermore, young animals showed a longer relative duration of lying than older ones (Figure 8b), *p* < .05 for young versus subadult, *p* < .01 for subadult vs. adult, *p* < .0001 for young vs. adult, Wilcoxon test.

### 3.3 Lying—head down

In order to investigate the duration and structure of LHD phases, thus the time animals spend in the REM sleep position, we investigated the number of LHD phases per lying phase, the total duration of LHD during a lying phase, respectively its proportion, as well as the typical duration of a LHD phase.

**FIGURE 9:**
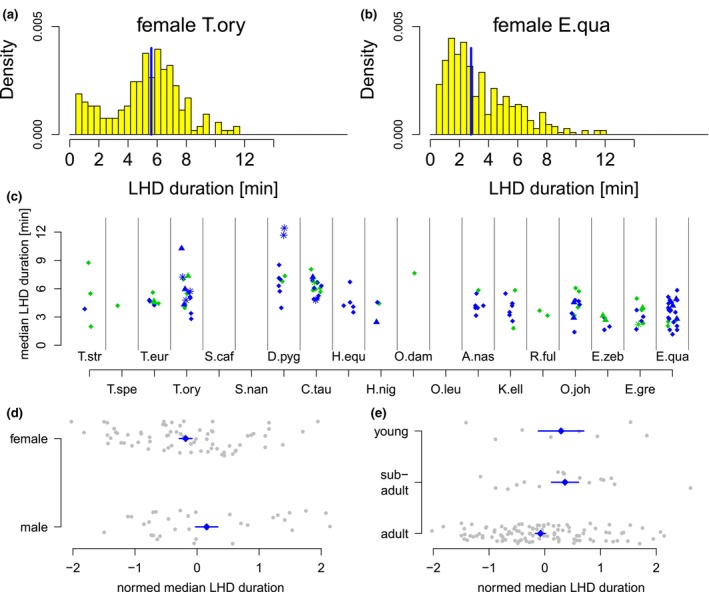
Duration of LHD during lying phase. (a, b) Examples of two distributions of LHD durations for a female Common Eland (a) and a female Plains Zebra (b). The distributions are nonsymmetric. (c) Median LHD duration as a function of the species. Point characters and colors as in Figure 6. (d) Species‐standardized LHD duration as a function of sex. (e) Species‐standardized LHD duration as a function of age group. Blue points and lines in (d, e) indicate mean and standard error, respectively.

Figure 9a,b show examples of LHD length distributions of a female Common Eland and a female Plains Zebra. Most distributions did not tend to be symmetric. We, therefore, used the medians (blue vertical lines) as a measure of location. Figure 9c shows the median LHD durations for all individuals for which nights with LHD could be recorded. The mean median LHD duration per species ranged from about 2.2 min in adult Mountain Zebras to about 7.6 min in adult Blesboks.

In order to investigate differences between sex and age groups, we again applied species standardization, using the mean and standard deviation of median LHD durations of all adult individuals per species for standardization. Figure 9d,e show the results as a function of sex and age, respectively. Females tended to show slightly smaller median LHD durations than males, where this difference was not significant at the 5% level. The group of young and subadult animals showed a slightly larger median LHD duration as compared to the adult individuals of the same species (.05 < *p* < .1, Wilcoxon test).

**FIGURE 10:**
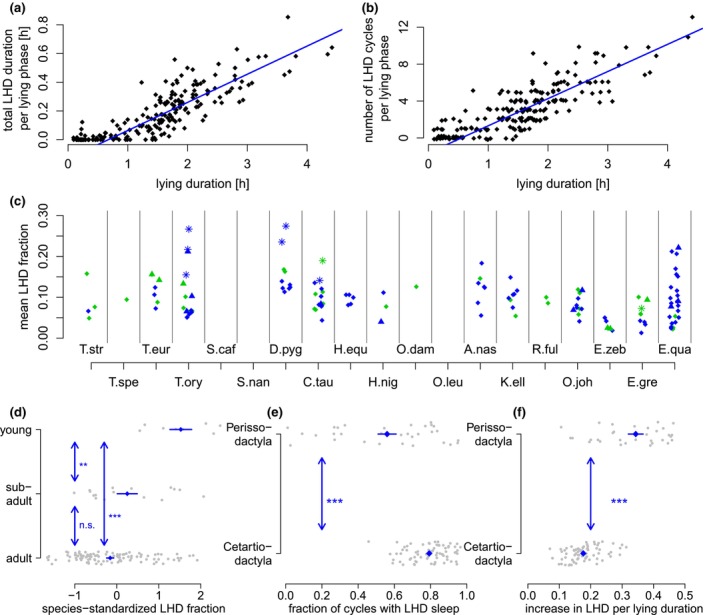
Relative duration of LHD during lying phase. (a) The LHD duration increases linearly with the length of the lying phase. Example of a female adult Waterbuck. Blue line indicates censored linear regression line, censored at zero because negative LHD durations cannot be observed. (b) The number of LHD cycles per lying cycle increases linearly with the length of the lying phase. Example of the same female adult Waterbuck. Blue line indicates censored regression line, censored at zero. (c) Mean LHD fraction (LHD duration divided by duration of lying phase) as a function of the species. Point characters and colors as in Figure 6. (d) Species‐standardized LHD fraction as a function of age group. (e) Increase in LHD duration per Lying duration (slope of (a)) for Zebras and animals of other species (only adult animals). (f) Fraction of observed cycles with LHD sleep for Zebras and animals of other species (only adult animals). Stars indicate statistical significance on the 5% (*), 1% (**) and 0.1% (***)‐level.

The number of LHD phases and its total duration seemed to increase linearly with the duration of the lying phase (Figure 10a,b show an example of a female adult Waterbuck), suggesting a certain regularity in the LHD structure with a roughly constant degree of LHD throughout the lying phase. Blue lines indicate censored regression lines where points are assumed to be censored at zero, as the number of LHD phases and the total time spent in LHD cannot be negative. Across all animals, no systematic differences in the intersection of the censored regression line (Figure 10a) with the *x*‐axis could be observed, indicating no systematic differences in the minimal lying duration that is required for LHD. The median minimal lying duration such that LHD was observed was 36.7 min. Notably, the increase in LHD duration per lying duration (slope in Figure 10a) showed no systematic age differences. Slopes tended to be higher in zebras (Figure 10e) than in other species, suggesting a higher increase in LHD duration per lying duration. However, zebras showed more lying phases without LHD (Figure 10f, *p* < .1%, Wilcoxon test between adult zebras and adult individuals of the order Artiodactyla). No further systematic differences in the fraction of lying cycles with LHD could be observed as a function of sex or age.

Concerning the proportion of LHD during a lying phase, that is, total LHD duration per phase divided by length of lying phase, we observed no systematic differences across species (Figure 10c). However, the proportion of LHD per lying phase tended to be larger in young animals (Figure 10d, LHD fraction standardized per species, Kruskal‐Wallis‐Test *p* < .1% for all three age groups, *p* < .1% for young vs. subadult, and *p* < .1% for young vs. adult animals, subadult vs. adult not significant). In particular, adult individuals (across all species), spent 9.9% of a lying phase in the LHD position while this value was 19.4% for juvenile and 10.3% for subadult individuals.

## 4 DISCUSSION

### 4.1 Review of the results

In this contribution, rhythms in the nocturnal behavior of ungulates have been studied. Most of the analyzed species showed a significant increase in the proportion of lying during the second phase of a night, some species additionally showed an increase during the first part of the night. The duration of the LC was approximately Gaussian for most individuals, with respect to species, age or sex. The lying fraction LF varied between all age groups and was found to be greater in younger animals. In general, zebras showed a lower LF than Artiodactyla. No differences were found in the proportion of LC without LHD as a function of age or sex, but zebras had a lower proportion of such LC than the studied Artiodactyla. The minimum duration of a lying phase before LHD occurred did not vary systematically with age, sex or species. Although the fraction of LHD per LC was greater in young animals and the increase in time spent with LHD per lying phase was greater in Perissodactyla than in Artiodactyla, no species specific differences were found with respect to the proportion of a typical lying phase spent in LHD between adult individuals. More precisely, adult individuals spent, in the mean, 9.1% of a typical lying phase in LHD. Finally, the average median LHD duration ranged between 2.2 min in Mountain Zebras to about 7.6 min in Blesboks. Moreover, male individuals were found to have slightly longer LHD phases than females.

### 4.2 Differences between sex and age groups

We analyzed differences in the lying cycles, the lying fraction and the time spent in the REM sleep position (LHD) as a function of the age and sex of the individual, and we graphically investigated differences between species.

Differences between age groups were most prominent. In particular, younger animals showed a high proportion of extremely short lying cycles. The lying fraction, that is, the proportion of lying in a lying cycle, of younger individuals was greater than the lying fraction of adult individuals. This is in accordance with previous studies which have shown that activity/rest cycles may vary as a function of age (Ruckstuhl & Neuhaus, 2009; Siegel, 2005; Steinmeyer et al., 2010). In addition, we observed differences in the median time spent in the REM sleep position as a function of age. Specifically, young and subadult animals tended to spend more time in the REM sleep position during a typical lying phase than adult individuals. Statistically, this difference was not significant on the 5% level but showed a trend with *p* < .1. Furthermore, this extends results of a previous study on the same dataset (Gubert et al., 2023), which showed systematic differences of REM sleep duration between different age groups, while the total time spent in LHD decreased with increasing age. Similarly, the observation that the REM sleep patterns vary as a function of the age of the individual corresponds to similar findings in various species in mammals and birds (Cajochen et al., 2006; Rattenborg et al., 2017; Ruckstuhl & Kokko, 2002; Steinmeyer et al., 2010).

We could replicate small, but statistically nonsignificant, sex specific differences in the median length of a phase spent in the REM sleep position. This is in agreement with other reports, in which some observed similarly that males tended to stay longer in the REM position, as reported in a case study on Common Eland behavior (Gubert et al., 2022), or studies of other mammals and birds (Cajochen et al., 2006; Rattenborg et al., 2017; Steinmeyer et al., 2010). It is also in agreement with studies which suggest that sex differences are small or occur only in dissimilar‐sized species (Ruckstuhl & Kokko, 2002), or are not considered as a possible or significant factor at all (Burger et al., 2021; Gubert et al., 2023; Tobler & Schwierin, 1996; Zhang, 2000).

Moreover, we observed several differences between the three zebra species as Perissodactyla and all other species as Artiodactyla. In particular, the Perissodactyla showed a much lower proportion of lying per lying cycle. This fits well with previous findings, for example, with a study on farm animals, which suggested that the total time spent lying is much lower in Equidae than in Bovidae (Ruckebusch, 1972). Moreover, similar effects were observed in the previous study on this dataset (Gubert et al., 2023). The reason behind this observation might be found in the different digestion types. Indeed, zebras, which are hind‐gut fermenters, require a larger food intake than ruminants (Owen‐Smith & Goodall, 2014). In particular, this leads to increased foraging behavior. Ruminants, on the other hand, spend more time in a lying position because ruminating occurs often while resting (Janis, 1976). The current results extend the previous findings, as they do not only refer to the proportion spent with a specific behavior during the night, but they show that the time spent in the lying position is well distributed over the night. Furthermore, the increase in the fraction spent in the REM sleep position per lying phase is greater within Perissodactyla. This, together with the lower overall lying fraction, suggests that they also have more periods of lying without being in the REM sleep position at all. Finally, the mean duration of an LHD phase in adult zebras tended to be smaller, ranging from 2.2 min in Mountain Zebras to about 7.6 min in Blesboks. These durations fit well with the literature. Lesser mouse‐deers (*Tragulus kanchil*) were observed to spend 2.0 ± 0.2 min in the REM sleep position (Lyamin et al., 2021), adult Common Elands had a median REM duration of 4.4–4.6 min (Gubert et al., 2022), and male Arabian oryx spent 7 ± 2 min in the REM sleep position in the dark in winter (Davimes et al., 2018). Moreover, the longest phases per night spent in the REM sleep position were observed to be 6.6 ± 4.0 min for horses (*Equus* sp.) (Pedersen et al., 2004) and the corresponding average phase length was found to be 3.9 min (Ruckebusch, 1972).

### 4.3 Trends and rhythms

Despite all the differences discussed above, many similarities could be observed between all individuals, regardless of species, age, or sex. All individuals showed a consistent behavior during most nights, see Figure 3 for an example. Furthermore, there was an increase in the proportion of lying during the second half of a night found in all but three species. Similar observations have been made for some species such as Arabian Oryx, Common Elands, Blue Wildebeests or African Elephants, where inactivity increases during the night (Clauss et al., 2021; Davimes et al., 2018; Gravett et al., 2017; Gubert et al., 2022; Malungo et al., 2021). Okapi, Plains Zebra, Common Eland and Common Wildebeest also showed an increase in the first part of the night. Those species have in common that they are larger and more fortified than most other analyzed species. The three exceptions that did not show a significant increase in the second part of the night also have a visual increase until the last 1–2 h. A possible explanation is that the behavior is just shifted by a few hours, and over a 24‐h cycle these species could fit well into one of the two clusters.

In addition, all species show a similar mean lying cycle duration of about 2.14 h, that is, typically an individual lies down every 2.14 h. Lying cycle duration follows a Gaussian distribution for most individuals and is well concentrated. This implies that there is a high degree of rhythmicity in the nocturnal behavior of the studied ungulates. Deviations from this rhythmicity could, therefore, be a good indicator of reduced animal welfare, but further studies would be needed to examine the influence of disturbing events on this rhythmicity.

### 4.4 Implications with regard to REM sleep

One focus of the current study was to describe and analyze periods spent in the REM sleep position. We found the average length of such a period to range from 2.2 min (Mountain Zebra) to 7.6 min (Blesbok). This fits well into the sparse existing literature, as in general, ungulates spend only short periods of time in the REM sleep position (Davimes et al., 2018; Ruckebusch, 1972). It is to notice that REM sleep plays an important role in several physiological processes (Blumberg et al., 2020). Therefore, events that reduce the duration of REM sleep could have a negative impact on an animal's wellbeing (Mellman et al., 2002; Sicks, 2016; Siegel, 2001; Suchecki et al., 2012). Such events could be perturbations that reduce the duration of REM sleep in the long term, or stress events that have only a short impact. Field studies show that environmental conditions can strongly influence the timing of sleep. For example, seasonal variation affects REM sleep in free‐ranging Arabian Oryx. Particularly, Arabian Oryx spent much more time in REM sleep during winter, than they do in summer (Davimes et al., 2018). Also, extreme weather events such as strong thunderbolts prevent Giraffes from lying down, which shortens the total duration of sleep (Burger et al., 2020). In addition, some studies suggest an influence of predation risk on the length and timing of REM sleep, that is, a higher predation risk indicates shorter typical REM sleep phases (Allison & Cicchetti, 1976; Lima et al., 2005). One reason for this effect may be that large terrestrial animals, such as many ungulates, must lie down during REM sleep due to loss of muscle tone, which increases their vulnerability to predation (Lima et al., 2005; Ternman et al., 2014). Our results suggest that a minimum amount of lying time is usually required for an animal to be in the REM sleep position at all and that the time spent in the REM sleep position, as well as the number of such phases during a lying phase, increases linearly with the length of the lying event (see Figure 10). Therefore, it is natural to ask whether the aforementioned external factors may affect animals in such a way that they cannot lie down long enough to show REM sleep. Future comparative studies of zoo and wild animals could, for example, focus on whether the environment of zoos, without the risk of predation, is associated with changes in REM sleep patterns.

## AUTHOR CONTRIBUTIONS

**Jennifer Gübert:** Conceptualization (lead); data curation (lead); formal analysis (supporting); investigation (lead); methodology (lead); visualization (supporting). **Gaby Schneider:** Data curation (supporting); formal analysis (lead); funding acquisition (equal); visualization (lead); writing – original draft (equal). **Max Hahn‐Klimroth:** Data curation (supporting); formal analysis (supporting); visualization (supporting); writing – original draft (equal). **Paul W. Dierkes:** Conceptualization (supporting); funding acquisition (equal); resources (lead); supervision (lead); writing – original draft (equal).

## FUNDING INFORMATION

The study received financial support from von Opel Hessische Zoostiftung and LOEWE Schwerpunkt CMMS—Multiscale Modeling in the Life Sciences.

## CONFLICT OF INTEREST STATEMENT

The authors declare that the research was conducted in the absence of any commercial or financial relationships that could be construed as a potential conflict of interest.

## Supporting information


Appendix S1.

## Data Availability

The original contributions presented in the study are included in the article/Appendix S1, further inquiries can be directed to the corresponding author/s.
